# Case of Mento-Posterior Impaction:—Successful Raising and Rotation of Head

**Published:** 1895-09-14

**Authors:** Glynn Whittle

**Affiliations:** Senior Physician to the Liverpool Lying-in Hospital


### CASE OF MENTO-POSTERIOR IMPACTION:—SUCCESSFUL RAISING AND ROTATION OF HEAD.

By GLYNN WHITTLE, M.A., M.D.Cantab, Senior Physician to the Liverpool Lying-in Hospital.

This was a face presentation, the chin being directed backwards. It was one of those cases in which, as has been forcibly remarked, "Nature is hopelessly condemned and art must intervene." The mode of procedure, which was finally adopted with success, at first appeared to me to be the least hopeful course. I therefore made an effort to deliver by a manœuvre recommended by several eminent authorities, viz., the proceeding, whose description follows, and which has three separate stages. The first stage is the application of the forceps, so as to grasp the head in its transverse diameter. The second consists in gradually pulling the head lower and lower, making the soft parts of the mother yield, until the face protrudes through the vulva, and the chin is forcibly slipped past the perineum. The third stage is the combination of traction with a smart turn of the handles backwards round the coccyx, the effect being to flex the head and successively deliver the forehead and cranium. It became evident, however, that it would be impossible for me to complete the second stage of this mode of delivery without grave risk to the mother and the probable sacrifice of the child. I therefore had to abandon the attempt, but, before resorting to craniotomy, it occurred to me that possibly the head might be less hopelessly jammed in the pelvis than it appeared to be. I therefore commenced to push the head (held in the grasp of the forceps) upwards, and with strong pressure very steadily maintained gradually succeeded in raising it till I was hopeful that the pelvis would no longer obstruct possible rotation. I then attempted and slowly accomplished the rotation of the head in a semicircle, marked lateral pressure on the wrist being occasioned by the effort. Subsequently, as far as the long forceps with their sacral concavity now directed backwards would permit, I drew down the head so as to fix it in the favourable position. The next step was to withdraw the forceps from their awkward position, and to reapply them, sacral convexity backwards, or in other words in their usual position. Flexion was thus assisted, and safe delivery, favourable to both mother and child, followed in due course. These chin-backwards face presentations, thus treated, are rare, this patient being the only case of the kind which has occurred in the Liverpool Lying-in Hospital since it was rebuilt and its medical staff reconstructed ten years ago.

